# Paeonol Inhibits Proliferation of Vascular Smooth Muscle Cells Stimulated by High Glucose via Ras-Raf-ERK1/2 Signaling Pathway in Coculture Model

**DOI:** 10.1155/2014/484269

**Published:** 2014-06-05

**Authors:** Junjun Chen, Min Dai, Yueqin Wang

**Affiliations:** ^1^Key Laboratory of Xin'an Medicine, Ministry of Education, Anhui Province Key Laboratory of R&D of Chinese Medicine, Anhui University of Traditional Chinese Medicine, Hefei 230038, China; ^2^School of Pharmacy, Anhui University of Traditional Chinese Medicine, Shihe Road 45, Hefei, Anhui 230031, China

## Abstract

Paeonol (Pae) has been previously reported to protect against atherosclerosis (AS) by inhibiting vascular smooth muscle cell (VSMC) proliferation or vascular endothelial cell (VEC) injury. But studies lack how VSMCs and VECs interact when Pae plays a role. The current study was based on a coculture model of VSMCs and VECs to investigate the protective mechanisms of Pae on atherosclerosis (AS) by determining the secretory function of VECs and proliferation of VSMCs focusing on the Ras-Raf-ERK1/2 signaling pathway. VECs were stimulated by high glucose. Our data showed that high concentration (35.5 mM) of glucose induced damage in VECs. Injury of VECs stimulated VSMC proliferation in the coculture model. Pae (120 **μ**M) decreased vascular endothelial growth factor (VEGF) and platelet derivative growth factor B (PDGF-B) release from VECs and inhibited overexpression of Ras, P-Raf, and P-ERK proteins in VSMCs. The results indicate that diabetes modulates the inflammatory response in VECs to stimulate VSMC proliferation and promote the development of AS. Pae was beneficial by inhibiting the inflammatory effects of VECs on VSMC proliferation. This study suggests the inhibitory mechanism of Pae due to the inhibition of VEGF and PDGF-B secretion in VECs and Ras-Raf-ERK1/2 signaling pathway in VSMCs.

## 1. Introduction

Atherosclerosis (AS) is a major pathological disease for cardiovascular and cerebrovascular problems and also the most common disease in the cardiovascular system [1]. AS is harmful to human health seriously and responsible for most of the deaths in the senior population [2]. Diabetes mellitus is considered as an important risk factor to the accelerated atherosclerosis [3–5]. High glucose has been shown to injure vascular endothelial cells (VECs) and vascular smooth muscle cells (VSMCs), which are two important cells of artery wall and responsible for AS progression [6]. Among them, VEC inflammatory injury and dysfunction are initial factors, and VSMC proliferation and migration are key pathological features [7–9]. In the native artery, VSMCs are tightly associated with VECs in structure and function. VECs dysfunction will release cytokines like VEGF, bFGF, TGF-*β*, and PDGF which have shown regulatory effects on VSMC proliferation and migration.

The mitogen activated protein kinase (MAPK) signaling pathway is one of the most important pathways for cellular stress response to injury. The MAPK signaling pathway consists of four subfamilies: p38 kinases, Jun-NH2-terminal kinases (JNK1/2), extracellular signal-regulated kinases (ERK1/2), and ERK5 [10]. Signaling through ERK1/2 is typically initiated by Ras, which can be activated by cytokines like VEGF and PDGF-B [11]. They activate Ras, which directly couples with the Raf (MAPK kinase kinase) and then combines with MEK1/2 (MAPK kinases). MEK1/2 functions as a dual specific kinase, which phosphorylates ERK1/2 directly. High concentrations of glucose (GS) have been shown to induce VEC membrane damage by increasing the release of inflammatory cytokines, such as vascular endothelial growth factor (VEGF) and platelet derived growth factor-B (PDGF-B) which bind with VSMC membrane receptors and lead to the receptor phosphorylation [12–14]. The activated receptors stimulate downstream signal transduction pathways and eventually activate ERK1/2 protein which lead to VSMC proliferation and intimal thickening [15, 16]. Previous studies have shown that PDGF-B expression was increased when VECs were damaged [17]. PDGF-B then activates the ERK1/2 related signaling pathway, which mainly mediates growth factor-induced cell proliferation [14, 18, 19]. Statistical analysis also showed that VEGF increased in diabetics and associated with blood glucose concentration which can also activate signal pathway involved in ERK1/2 participate [20]. Accordingly, we hypothesise that high glucose induced VECs producing a series of biologically active substances which stimulated VSMC proliferation.

**Figure 1:**
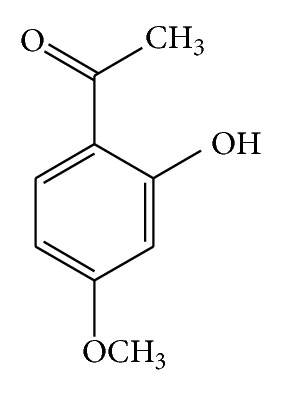
Chemical structure of paeonol.

Paeonol (Pae, 2′-hydroxy-4′-methoxyacetophenone, Figure 1) is the major biologically active compound contained in* Cortex Moutan* (*Paeonia suffruticosa* Andrews, Ranunculaceae), which is a Chinese herbal remedy widely used in clinical treatment of inflammatory diseases such as atopic dermatitis, hyperlipidemia, and atherosclerosis [21, 22]. Our previous investigations suggested that Pae had a significant effect on different aspects of AS.* In vivo*, Pae prevented AS in our experimental model and protected against arterial endothelial cell hyperlipidemia [21, 23, 24].* In vitro*, serum containing Pae significantly inhibited TNF-*α*-induced VSMC proliferation. Furthermore, our most current research confirmed that Pae significantly reduced the phosphorylation levels of JNK1/2, p38, and ERK1/2, which was activated by TNF-*α*, ox-LDL, and/or bacterial lipopolysaccharide in VECs [25]. Unfortunately, the previous* in vitro* studies were based only on a single cell type (VECs or VSMCs) and ignored the interactions between these two important cell types.

The current study was focused on a coculture model to determine essential crosstalk pathways between VECs and VSMCs. The coculture model is a novel method which could simulate the environment in native artery to study Pae action. The principal aim of this study was to investigate the effects of Pae on VECs cellular damage and its downstream effects on VSMC proliferation, which ultimately leads to the pathological feature observed in AS. In addition, this study worked to determine the relationship between the secretion function of damaged VECs and the Ras-Raf-ERK1/2 signaling pathway in VSMCs in order to clarify the therapeutic mechanisms of Pae. This experiment model provided a theoretical basis for Pae intervention in AS and optimized a technical platform to determine the cellular target of novel therapeutic compounds.

## 2. Materials and Methods

### 2.1. Chemicals and Reagents

The compound paeonol (99% purity) was obtained from Baicao Plants Biotech Co., Ltd. (Anhui, China). Dulbecco's modified Eagle's medium (DMEM), Transwell chamber, type I collagenase, and fetal bovine serum (FBS) were purchased from Gibco Life Technologies, Co., Ltd. (Paisley, UK). 3-(4,5-dimethylthiazolyl-2)-2,5-diphenyltetrazolium bromide (MTT) was obtained from Sigma Chemical Co. (St. Louis, MO, USA). Lactic dehydrogenase (LDH) reagent was purchased from Nanjing Jiancheng Bioengineering Institute (Nanjing, China). Antibodies against PDGF-B, Ras, Raf, phosphorylated Raf (P-Raf), ERK1/2, and phosphorylated ERK1/2 (P-ERK1/2) were obtained from Cell Signaling Technology (Beverly, MA, USA). PDGFR inhibitor (Sunitinib Malate) and ERK1/2 inhibitor (PD98059) were purchased from Santa Cruz Biotechnology Co. (Santa Cruz, CA, USA).

### 2.2. Animals

Sprague-Dawley (SD) rats (160 ± 10 g) were obtained from Shanghai Super-B&K Laboratory Animal Corp. Ltd. (license number: SCXK 2008-0016). All animal protocols were conducted in accordance with animal welfare protocols at the local institution Animal Care and Use Committee.

### 2.3. Cell Culture

VECs and VSMCs were isolated from rat thoracic aortas by primary explants techniques according to a previously published protocol [26, 27]. Briefly, the cells were incubated in a 50 mL culture flask at 37°C in a humidified atmosphere containing 5% CO_2_. Culture medium was composed of DMEM supplemented with 20% FBS, NaHCO_3_ (1.8 g/L), penicillin 100 kU/L, and gentamicin 100 kU/L. The culture media were changed every 3 d. Cells were grown to an 80% confluence state and subcultured using 0.2% trypsin. VECs and VSMCs at passages three to five were used in the current study.

### 2.4. VECs and VSMCs Coculture

The coculture model was created to investigate the effects of damaged VECs on VSMCs through polycarbonate filter membrane (Transwell chamber). The coculture model was created according to the methods of Fillinger et al. [28, 29]. VECs and VSMCs were diluted into cell suspension of 1 × 10^5^ cells/mL. VECs were inoculated into the bottom of a 6-well chamber and pretreated with a high glucose (HG) concentration (35.5 mM) for 48 h, whereas 5.5 mM was considered as normal glucose concentration. Then, VSMCs were inoculated into the top of the Transwell plate. The Transwell chamber was then set into the 6-well chamber and cocultured. The two types of cells were not physically connected but were able to interact by secreting soluble factors through a polycarbonate filter membrane.

### 2.5. Cell Survival Rate Assay

The cytotoxic effects of glucose and Pae on VECs growth were determined through the 3-(4,5-dimethylthiazolyl-2)-2,5-diphenyltetrazolium bromide (MTT) assay. VECs were grown to 80% confluence and then seeded into a 96-well flat-bottom plate and incubated with DMEM supplemented with 20% FBS. Different concentrations of glucose (5.5, 15.5, 25.5, 35.5, and 45.5 mM) at multiple time points (0, 12, 24, 48, and 72 h) were used. Furthermore, to investigate the effects of Pae on VECs, cells were pretreated with different concentrations of Pae (7.5, 15, 30, 60, and 120 *μ*M) for different time points (0.5, 6, 12, and 24 h) before being stimulated by glucose at a suitable concentration (35.5 mM) for 48 h. Cells were incubated with glucose at 5.5 mM, which is indicated as the normal glucose (NG) group. After treatment, cells were incubated with MTT (20 *μ*L/well) (Sigma Chemical, USA) for 4 h at 37°C. The medium was then removed and DMSO (200 *μ*L/well) was added to solubilize the precipitate. The absorbance then was measured at 490 nm on an absorbance microplate reader (Molecular Devices, USA).

### 2.6. Lactic Dehydrogenase (LDH) Release Assay

The LDH release was used to investigate the cytoprotective effects of Pae on VEC injury due to high glucose concentrations. VECs were centrifuged at 160 ×g for 8 min to obtain a supernatant comprised of extracellular VEC components. VECs were incubated with 2% Triton X-100, freezed and thawed three times, and centrifuged at 240 ×g for 5 min to obtain a supernatant comprised of intracellular components. LDH concentrations in the extracellular medium and intracellular medium were quantified through the LDH reagent for clinical diagnosis.

### 2.7. Immunocytochemistry Assay

VECs seeded on coverslips were rinsed with ultrapure water twice, fixed with acetone for 20 min, and then rinsed with phosphate-buffered saline (PBS). Cells were soaked in 3% H_2_O_2_ at room temperature for 30 min. After washing with PBS, blocking buffer containing 1% goat serum was added and incubated for 20 min. The primary antibody (1 : 200 diluted) against VEGF or PDGF-B was added and incubated at 4°C overnight. A biotinylated secondary antibody was incubated for 20 min; then Streptavidin/Peroxidase (SP) reagent was incubated for 20 min at room temperature. The slides were colored with DAB under a light microscope for 10 min before examination. Immunocomplexes were visualized by the DAB detection system. Brown or dark brown stained cells were considered positive cells. Ten randomly selected fields were visualized at 200x magnification.

### 2.8. Western Blotting Analysis

Cytoplasmic proteins were obtained using a cell lysis buffer (20 mM HEPEs, 2 mM MgCl_2_, 1 mM EDTA, 2 mM DTT, 1 mM PMSF, pH 7.4) and stored at −80°C. The protein concentrations were quantified by the BCA method. Aliquots (30 *μ*L) were separated on a 10% SDS-PAGE and transferred to an equilibrated polyvinylidene difluoride membrane (PVDF) by electroblotting. Membranes were blocked in 5% fat-free milk for 2 h at room temperature and incubated at 4°C overnight with primary antibodies (rabbit anti-Ras, rabbit anti-Raf, rabbit anti-phospho-Raf, mouse anti-ERK1/2, and rabbit anti-phospho-ERK1/2). Horseradish peroxidase labeled secondary antibodies were added and incubated at room temperature for 2 h. Bands were detected by enhanced chemiluminescence (ECL) kit. Beta-actin protein levels were used as an endogenous control to allow the normalization of target proteins. The band intensity was quantified and analyzed with Quantity One software (Bio-Rad, USA). The results were quantified by using the integrated optical density of each band with the background subtracted.

### 2.9. Statistical Analysis

Data were analyzed by a one-way ANOVA or independent *t*-test. Data are presented as mean ± S.D. averaging three or more independent experiments. Significance was noted at a *P* < 0.05.

## 3. Results

### 3.1. High Glucose Injured VECs

**Figure 2:**
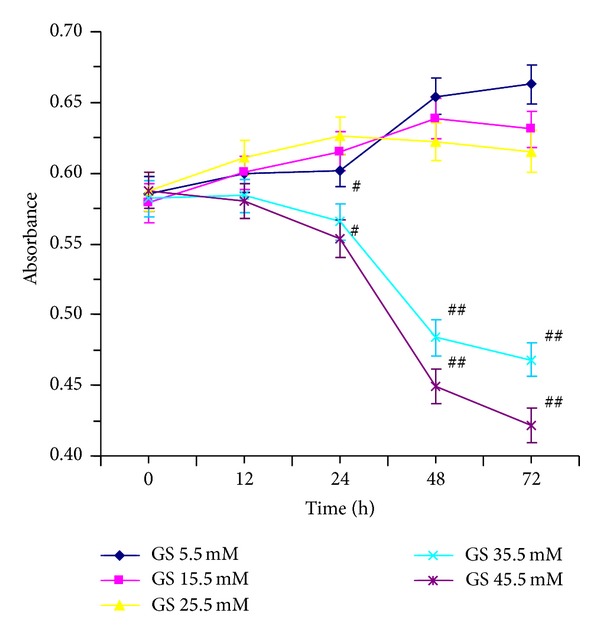
Effect of high glucose on VECs injury. VECs were induced by various concentrations (5.5, 15.5, 25.5, 35.5, and 45.5 mM) of glucose and incubated for different time points (0, 12, 24, 48, and 72 h) to explore the effect of glucose on VECs.

VEC survival was measured by MTT assay. VEC survival rates in glucose concentrations of 5.5 and 15.5 mM increased in a dose- and time-dependent manner. When the concentration was up to 25.5 mM, VEC survival rate was a little inhibited after incubatation for 48 h. However, the rate was inhibited significantly in glucose concentrations of 35.5 and 45.5 mM even in 24 h incubation. VEC morphology changed into contraction, rounded and smaller in glucose concentrations of 35.5 and 45.5 mM. Moreover, cells cultured in glucose concentrations of 35.5 mM for 48 h showed the most significant inhibition (Figure 2).

### 3.2. High Glucose Induced VSMC Proliferation in the Coculture Model

**Figure 3:**
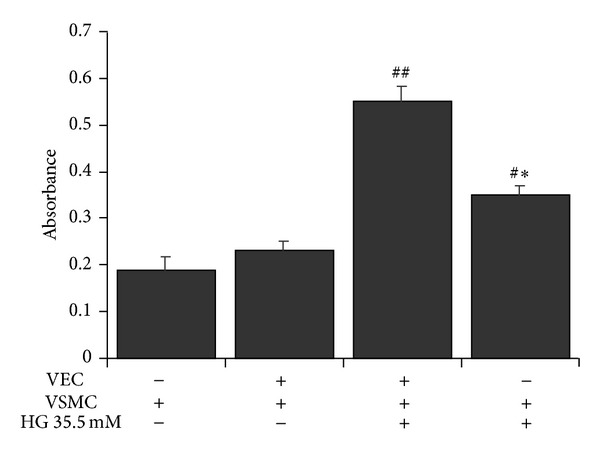
Effect of high glucose on VSMC proliferation in the coculture model. VECs were pretreated with high glucose concentration (35.5 mM) for 48 h and then cocultured with VSMCs for another 24 h to stimulate VSMC proliferation. ^#^ *P* < 0.05, ^##^ *P* < 0.01 versus NG (5.5 mM) coculture group; *P < 0.05 versus HG (35.5 mM) coculture group.

VECs were pretreated with a high glucose (HG) concentration (35.5 mM) for 48 h and then cocultured with VSMCs for 24 h to induce VSMC proliferation. Glucose concentration of 5.5 mM was considered as normal group. VSMCs inoculated in the upper chamber alone and stimulated with 35.5 mM glucose were set as the single cultured group. Compared with the normal group, VSMCs proliferated significantly in the cocultured group (*P* < 0.01), as well as in the single cultured group (*P* < 0.05). Compared with the single cultured group, VSMCs in the coculture group also proliferated significantly (*P* < 0.05), which suggested that injured VECs stimulated VSMCs proliferation (Figure 3). These results showed that VSMC proliferation stimulated by high glucose injured VECs was even much stronger than that stimulated directly by high glucose.

### 3.3. Paeonol Partially Restored Survival and Reduced LDH Release in VECs

**Figure 4:**
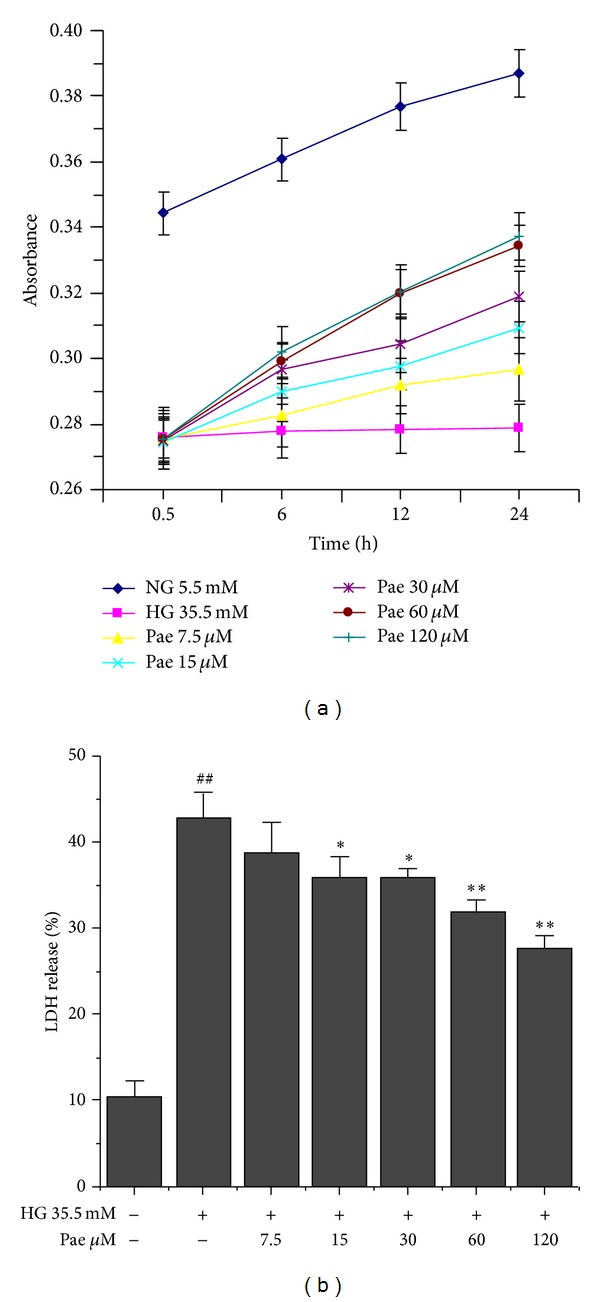
Effect of paeonol on survival and LDH release in VECs. VECs survival (a) and LDH release (b) induced by high glucose (35.5 mM) were determined by MTT assay. ^##^ *P* < 0.01 versus NG (5.5 mM) group; *P < 0.05, **P < 0.01 versus HG (35.5 mM) group.

VECs were pretreated with Pae at 7.5, 15, 30, 60, and 120 *μ*M for 24 h prior to being stimulated by high glucose. Compared with control group, VEC survival increased in a dose- and time-dependent manner. The effect of Pae was greatest (*P* < 0.01) when VECs were pretreated with Pae at 120 *μ*M (Figure 4(a)). Additionally, the LDH level was increased by the stimulation of 35.5 mM glucose. LDH release was gradually reduced as the Pae concentration increased. At a final concentration of 15 *μ*M Pae, LDH release was significantly decreased (*P* < 0.05). When the concentration of Pae was 60 *μ*M and 120 *μ*M, the difference in LDH release was highly significant (*P* < 0.01) (Figure 4(b)).

### 3.4. Paeonol Inhibited VEGF and PDGF-B Overexpression in VECs

**Figure 5:**
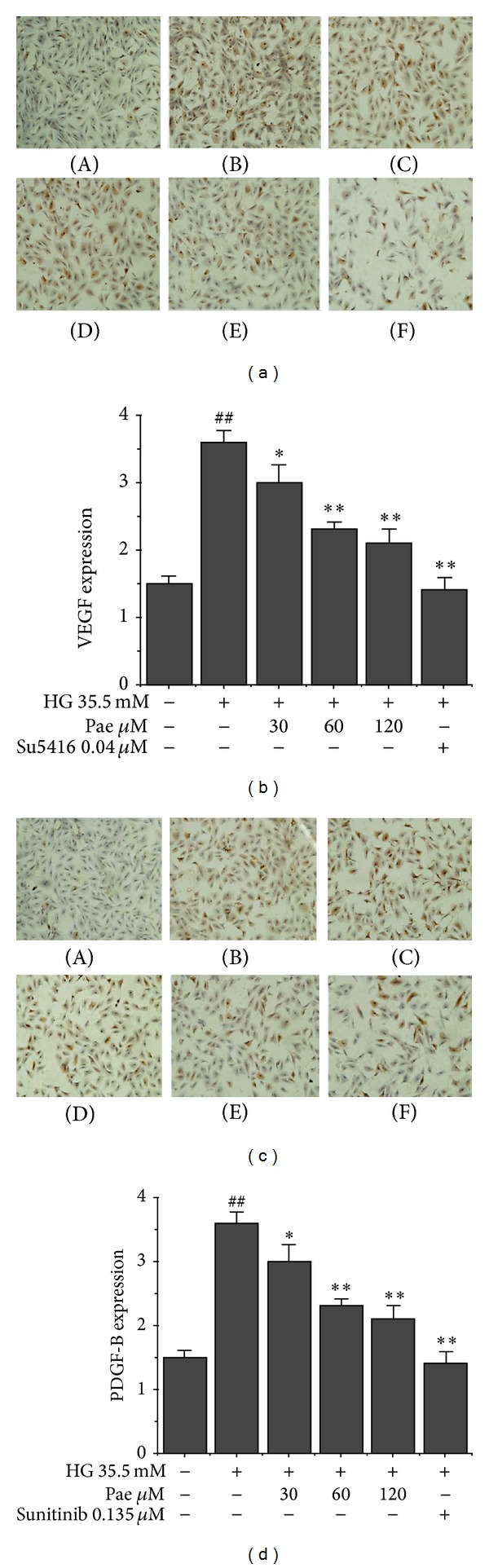
Effect of paeonol on VEGF and PDGF-B expression in VECs. VEGF (a and b) and PDGF-B (c and d) expression in VECs was performed through immunocytochemical staining as described in Section 2. (A) NG (5.5 mM); (B) HG (35.5 mM); (C) HG + Pae (30 *μ*M); (D) HG + Pae (60 *μ*M); (E) HG + Pae (120 *μ*M); (F) HG + Sunitinib (0.135 *μ*M). ^##^ *P* < 0.01 versus NG (5.5 mM) group; *P < 0.05, **P < 0.01 versus HG (35.5 mM) group.

Through immunocytochemical staining, VEGF and PDGF-B expression were quantified inside VECs. Positive cytoplasm was brown. The positive rate of VEC was used to evaluate the effect of high glucose and Pae. High glucose resulted in increasing expression of VEGF and PDGF-B in vascular endothelial cells. The expression decreased with increasing concentrations of Pae (Figures 5(a) and 5(c)). Concentrations of 60 *μ*M and 120 *μ*M Pae decreased the expression of VEGF and PDGF-B significantly (*P* < 0.01). As shown in Figures 5(b) and 5(d), the VEGFR inhibitor, SU5416, not only decreased the expression of VEGF significantly (*P* < 0.01) but also decreased the number of cells with brown nuclei. The PDGFR inhibitor, Sunitinib, showed the same results. These results suggested that the proliferation of VSMC induced by high glucose may be achieved by upregulating VEGF and PDGF-B factors. And Pae reversed the effect of high glucose. Based on the results, we chose an appropriate signaling pathway to further explore how the cytokine mediates proliferation of VSMC.

### 3.5. Paeonol Blocked Ras-Raf-ERK1/2 Pathway in VSMCs in Coculture Model

**Figure 6:**
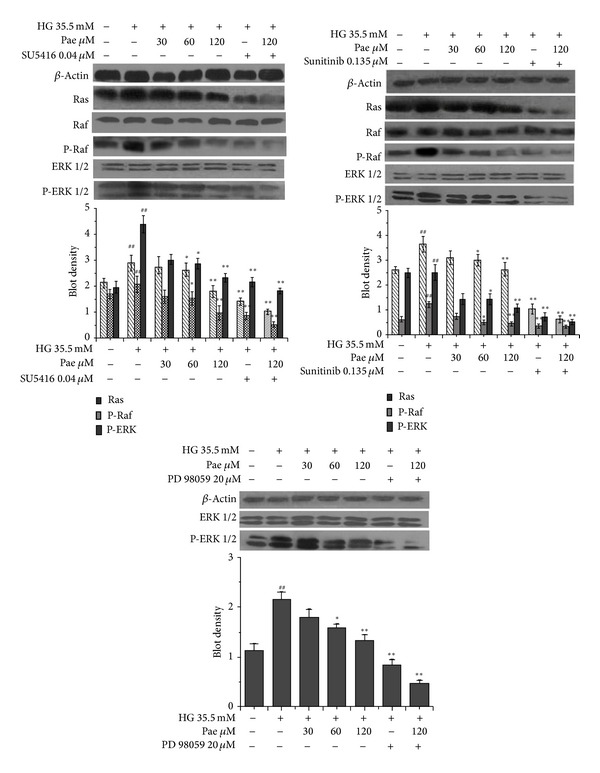
Effect of paeonol on Ras-Raf-ERK1/2 signaling pathway in VSMCs in the coculture model. VECs were pretreated with various concentrations of Pae for 24 h before being incubated with HG (final concentration of 35.5 mM) for another 48 h. Then VSMCs were inoculated into the top of the Transwell plate and cocultured with VECs for another 24 h. Protein concentrations were analyzed by Western blot. ^##^ *P* < 0.01 versus NG (5.5 mM) coculture group; *P < 0.05, **P < 0.01 versus HG (35.5 mM) coculture group.

One of the most important downstream signaling cascades of VEGF and PDGF-B is the Ras-Raf-ERK pathway [30]. To inhibit this signaling pathway initially, SU5416 and Sunitinib were used to inhibit the respective receptor and associated downstream proteins. In order to explore the cellular target of Pae, PD98059 was used to inhibit ERK1/2 protein expression. As shown in Figure 6, high glucose (35.5 mM) significantly induced the expression of Ras, P-Raf, and P-ERK1/2 in VSMCs (*P* < 0.01). Pae (120 *μ*M) significantly inhibited the expression of Ras, P-Raf, and P-ERK1/2 (*P* < 0.05). For protein Ras and P-Raf, SU5416 and Sunitinib significantly reduced the expression (*P* < 0.01). Likewise, cells treated with SU5416, Sunitinib, or PD98059 showed significantly inhibitory effect on P-ERK1/2 expression. Moreover, the cotreatment of Pae (120 *μ*M) with SU5416, Sunitinib, or PD98059 had a greater inhibitory effect than treating VSMCs with each individual compound. These results indicated that the effect of Pae to block Ras-Raf-ERK signaling pathway may be due to the ability to inhibit the expression of VEGF and PDGF-B.

### 3.6. Paeonol Inhibited VSMC Proliferation in Coculture Model

**Figure 7:**
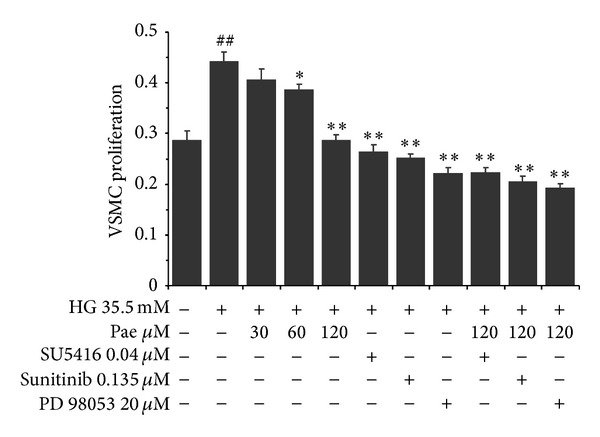
Effect of paeonol on VSMC proliferation in the coculture model. VECs and VSMCs cocultured model was established as described in Section 2. The effects of paeonol on VSMC proliferation were assayed by MTT. ^##^ *P* < 0.01 versus NG (5.5 mM) coculture group; *P < 0.05, **P < 0.01 versus HG (35.5 mM) coculture group.

VECs were pretreated with different concentrations of Pae (30, 60, and 120 *μ*M) before being stimulated by high glucose (35.5 mM) for 48 h. The results of MTT indicated that Pae (30, 60, and 120 *μ*M) inhibited VSMC proliferation (*P* < 0.01) compared with high glucose group. The levels of VSMC proliferation were also inhibited with Pae plus Sunitinib or PD98059 group. The level of inhibition was greater than each single treatment group, but no significant difference was observed (Figure 7). The results proved the protective effect of Pae on VSMC proliferation which cocultured with VECs in high glucose.

## 4. Discussion

Diabetes is one of the main predisposing factors of atherosclerosis. Type 2 diabetic vascular complications are based on diabetes-induced arteriosclerosis [31]. High glucose concentrations exerted a harmful effect on VECs and VSMCs, which are important components of artery wall. Impaired VECs destroy the structure of the endothelial monolayer and lead to vascular barrier dysfunction [32]. Large numbers of damaged VECs have been shown to impact the vasodilatation function and VECs regeneration [33]. These effects may be an important mechanism of diabetic complication of AS [34]. Our present study indicated that high glucose concentrations increased membrane permeability and decreased VEC survival. VSMC proliferation and phenotypic transformation are critical pathological features of AS, which depend on growth factors, cytokines, and vasoactive substances. VEGF is the initial factor for early VEC angiogenesis and VSMC proliferation. However, a mature vascular system can not be generated alone. PDGF-B also had a strong effect on promoting inflammation in this study. Several reports support the hypothesis that PDGF-B plays a crucial role in the development of atherosclerotic plaques [35–37]. Our study demonstrated that VEGF and PDGF-B expression in VECs was increased significantly by high glucose. Treatment with Pae significantly attenuated VEGF and PDGF-B expression. Therefore, our results indicate that Pae may act against VEC injury by reducing VEGF and PDGF-B release as an anti-inflammatory and antithromboembolic effect.

Monolayer cell culture, in many cases, is difficult to simulate the interaction between various types of cells* in vivo*. Coculture model can simulate the internal* in vivo *environment to observe important interactions between cells [38]. In addition to the morphological changes, a variety of angiogenesis-related gene expression changes occur in VECs cocultured with VSMCs [39, 40]. Thus, the coculture model in this study not only retained the cellular microenvironment of material and structural basis* in vivo* but also showed the advantages of controllability and macroscopic visibility of the cell culture. The significant proliferation of VSMCs in the coculture model confirmed that damaged VECs could stimulate VSMC proliferation and also indicated that basic interaction between VECs and VSMCs can be carried out smoothly. Accordingly, the coculture model provides the necessary conditions to explore the pathogenesis of atherosclerosis.

Ras-Raf-ERK1/2 signaling pathway was activated by high glucose concentration in our coculture model. The phosphorylated proteins regulate the target gene expression which promote cell proliferation [41, 42]. Our study indicated that Pae protected the integrity and survival rate of VECs and reduced the VEGF and PDGF-B release of VECs into the cocultured model to inhibit VSMC proliferation. We also presume that the effect may relate to the downstream signaling pathway in VSMCs. For this reason, the study used the VEGFR and PDGFR inhibitor to explore the possible targets of Pae. The results showed that Pae in combination with SU5416 or Sunitinib could further reduce the Ras, P-Raf, and P-ERK1/2 expression levels. In order to further clarify whether Pae had effect on protein ERK1/2 as well, the ERK1/2 inhibitor, PD98059, was utilized. We found that ERK1/2 expression levels were reduced when treated with Pae and the inhibitor. These findings indicated that Pae may decrease the VEGF and PDGF-B expression, resulting in protecting the VEC and inhibiting the downstream signaling pathway. Otherwise, Pae may inhibit the VSMC proliferation due to the direct inhibition of Ras-Raf-ERK1/2 pathway.

Our findings confirmed that Pae can inhibit the proliferation of VSMC cocultured with VECs induced by high glucose concentration. One reason is that Pae decreased the release of inflammatory cytokines, VEGF and PDGF-B, in VECs which combined with VSMC membrane receptors and led to phosphorylation of the receptors. The inhibited receptors blocked downstream signaling pathways, Ras-Raf-ERK1/2, which are responsible for VSMC proliferation. The other is that Pae inhibited Ras-Raf-ERK1/2 activation directly to suppress VSMC proliferation. Based on our study, Pae appears to be a promising inhibitor to AS.

## 5. Conclusion

Taken together, our findings indicated that the mechanism of Pae effects on AS might involve its sequential inhibition of VEGF and PDGF-B in VECs and the Ras-Raf-ERK1/2 signaling pathways in VSMCs.
